# Impact of parental separation or divorce on school performance in preterm children: A population-based study

**DOI:** 10.1371/journal.pone.0202080

**Published:** 2018-09-07

**Authors:** Simon Nusinovici, Bertrand Olliac, Cyril Flamant, Jean-Baptiste Müller, Marion Olivier, Valérie Rouger, Géraldine Gascoin, Hélène Basset, Charlotte Bouvard, Jean-Christophe Rozé, Matthieu Hanf

**Affiliations:** 1 INSERM, CIC 1413, Nantes University Hospital, Nantes, France; 2 Department of Child and Adolescent Psychiatry, Centre Hospitalier Esquirol, Limoges, France; 3 INSERM, UMR1094, Tropical Neuroepidemiology, Limoges, France; 4 Department of Neonatal Medicine, Nantes University Hospital, Nantes, France; 5 Réseau “Grandir Ensemble”, Nantes University Hospital, Nantes, France; 6 Department of Neonatal Medicine, Angers University Hospital, Angers, France; 7 Department of Neonatal Medicine, Le Mans Hospital, Le Mans, France; 8 SOS Prema (parents of French preterm children organization), Boulogne-Billancourt, France; Center of Pediatrics, GERMANY

## Abstract

The objective of this study was to quantify the possible decrease in school performance at five years of age in preterm children associated with parental separation or divorce, and to test whether this effect varies according to the child’s age at the time of the separation. This study included 3,308 infants delivered at < 35 weeks of gestation born between 2003 and 2011 who were enrolled in the population-based LIFT cohort and who had an optimal neurodevelopmental outcome at two years of age. These infants were evaluated by their teachers to assess their abilities and behavior when they had reached five years of age, using the Global School Adaptation (GSA) questionnaire. The mean GSA score was 50.8 points. Parental separations (assessed as parents either living together or living separately) were associated with a decrease in school performance at five years of age, although this was only the case for children who exhibited difficulties at school (3.7 points, p < 0.01). A decrease in school performance only occurred when parental separations took place between 3 and 5 years after the child’s birth. Parental separation was associated with a decrease in these children’s levels of motivation, autonomy, and manual dexterity. This study indicates that preterm infants of parents who had separated are particularly at risk of a lower scholar performance.

## Introduction

Parental separation has been associated with reduced cognitive development and educational performance[1–6]. Since parental separation can result in multiple negative effects, including perceived guilt, blame, stressors, and diminished resources for the children, it is not surprising that parental separation has also been reported to negatively affect a child’s motivation, engagement, and learning-related behavior in the classroom[6,7].

Although several studies have investigated the impact of the timing of the parental separation on scholastic performance, they have yielded discordant results. Most studies found that for younger children (i.e. those in elementary school) the adverse impact was more pronounced than for older children (i.e. those in high school)[4,8,9], suggesting that younger children may feel more anxious about abandonment and that they may be more likely to blame themselves[10]. However, others studies have found that divorce may adversely affect adolescents more than elementary school-age children[11], or that the child’s age did not alter the effect of divorce on academic achievement[12]. Lastly, very few studies have focused on the effect of parental separation on very young children.

One would expect that a negative effect of parental separation on school performance should also be apparent in children who were delivered preterm, in light of the vulnerability of this population. Preterm births are indeed associated with lower educational performances[13–15]. Preterm children are at risk of achieving lower cognitive test scores, and their level of immaturity is directly proportional to the mean cognitive scores at school age[13]. To our knowledge, the effect of parental separation on the school performance of children who were delivered preterm has not been investigated to date. The objective of this study was (i) to quantify the possible decrease in school performance at five years of age that is associated with parental separation or divorce in preterm children and (ii) to test whether this effect varies according to the child’s age at the time of the separation.

## Materials and methods

### Study population

The study population comprised surviving preterm infants enrolled in the Loire Infant Follow-up Team (LIFT), born at less than 35 weeks of gestation between January 2003 and December 2010. The LIFT network includes 24 maternity clinics in the Pays-de-la-Loire region (one of the 13 administrative regions of France) with the objective of screening for early clinical anomalies associated with preterm births and to provide specifically adapted care. The follow-up consisted of standardized visits by trained physicians at 3, 6, 9, 18, and 24 months as well as at 3, 4, 5, 6, and 7 years after the birth of the child. The children were evaluated at two and five years of corrected age to assess their neurodevelopmental outcomes. Written consent was obtained at the time of enrollment in the study. The cohort was registered with the French Data Protection Authority (“Commission National de l’Informatique et des Libertés”–CNIL) (n^o^ 851117).

### Selection of optimal infants at two years of age

At two years of age, the neurodevelopmental evaluation was based on a physical examination by a trained pediatrician, a psychomotor evaluation by a psychologist and/or a questionnaire completed by the parents. This evaluation aimed to assess whether the infants had an optimal or a non-optimal neurodevelopment. Only infants that were considered to be optimal at two years of age were included.

### Abilities and behavior of the children at five years of age

At five years of age, the evaluation was based on a questionnaire for the children’s teachers, the “Global School Adaptation” (GSA) score, which allows for assessment of the children’s abilities and behaviors in the classroom. This questionnaire has been used previously by the French ministry of education to investigate the performance of children in the French public school system. It has been validated as a screening tool for adverse neurodevelopmental outcomes in preterm children[16]. Indeed, for more than 89% of the children in our cohort, the GSA score was highly or moderately consistent with the IQ score. At the age of five years ± two months, the kindergarten teachers were asked by the parents to complete this questionnaire. It comprises 20 items, exploring five areas: language, transferable skills, socialization, motor skills, and number processing[16]. Each item corresponded to a score between 1 and 3 (S1 Fig). The total score was calculated by adding the points for the 20 items, and it hence ranged between 20 and 60. The higher the GSA score, the higher the school performance level.

### Parental separation

Information regarding marital status was binary, i.e. it was rated either as parents living together or as parents living separately. For parents who had separated, the earliest date at which they were reported to have separated was used. To investigate whether the effect of parental separation varied according to the time of the separation, three groups of infants were considered: infants with intact families, infants whose parents had undergone separation prior to the 24-month visit (included), and those whose parents had separated between the 36 and the 60-month visits (included). Parental separations that occurred after the 60-month visit were excluded.

### Adjustment variables

Adjustment variables comprised perinatal characteristics, socioeconomic data, and urbanicity of the residential municipality. The following perinatal characteristics were taken into account in the analyses: gestational age (three classes: 24 to 27 weeks, 28 to 31 weeks, and 32 to 34 weeks GA), gender, twinship, and birth weight. The birth weight Z-score was computed according to the Olsen standards[17], and it was considered as a four-class-categorical variable (< -1, between -1 and 0, between 0 and 1, and > 1). The socioeconomic data consisted of the socioeconomic level and eligibility for social security benefits for those with low incomes. The socioeconomic level took into account the parent with the more highly rated job according to a scale based on the official classification developed by the French Institute for Statistics and Economic Studies (INSEE). Lastly, the residential municipality was considered to be either urban or rural based on definitions developed by the INSEE. Municipalities were considered to be urban if there no buildings separated by a horizontal distance of more than 200 meters (i.e. if it was a continuously built-up area) and there were at least 2,000 inhabitants. All other municipalities were considered to be rural.

### Statistical analyses

Infants whose parents had separated and infants whose parents were living together were matched using an exact matching procedure. Indeed, the possible differences between characteristics of infants whose parents had separated and infants whose parents were living together could bias the estimation of the effect of the separation on their school performance (school performance could be highly influenced by the gestational age, birth weight, and socioeconomic status in particular). The matching was based on the following covariates: their gestational age and birth weight Z-scores (as categorical variables), gender, twinship, socioeconomic level, eligibility for social security benefits for those with low incomes, and urbanicity. Exact matching resulted in groups with different numbers of infants that had the same characteristics. Therefore, weights were used in the following models to ensure that the matched infants were weighted up to be similar. This matching procedure allows the possible bias due to the differences between infants with parents living together and those who had separated to be minimized.

The effect of separation on the GSA score was quantified using weighted quantile regressions. Quantile regression allowed for quantification of the effect of separation on conditional quantiles of the GSA score, thus providing a complete view of possible relationships between parental separation and the GSA score. By contrast, a linear regression only allows an overall effect of parental separation on the mean of the GSA score to be discerned. Moreover, the effect of parental separation was tested on each of the 20 items of the GSA questionnaire. To do so, a weighted generalized estimated equation (GEE) was considered for each item (response 1 versus responses 2 or 3). All of the models were adjusted for the GA and Z-scores for the birth weights as continuous variables. All of the statistical analyses were performed with R software[18]. Finally, a sensitivity analysis was performed with three additional adjustment variables to test the robustness of the results. These variables were: breast feeding only, severe neurological anomalies, and the duration of oxygen supply.

### Ethics approval

The LIFT cohort (Grandir ensemble en Pays de la Loire) is registered at the French data protection authority in clinical research (Commission Nationale de l’Informatique et des Libertés or CNIL, No. 851117). This study received the favorable opinion of an ethics committee (GNEDS, Groupe nantais d’éthique en santé). Written consent was obtained from the parents of each child before inclusion.

## Results

**Fig 1:**
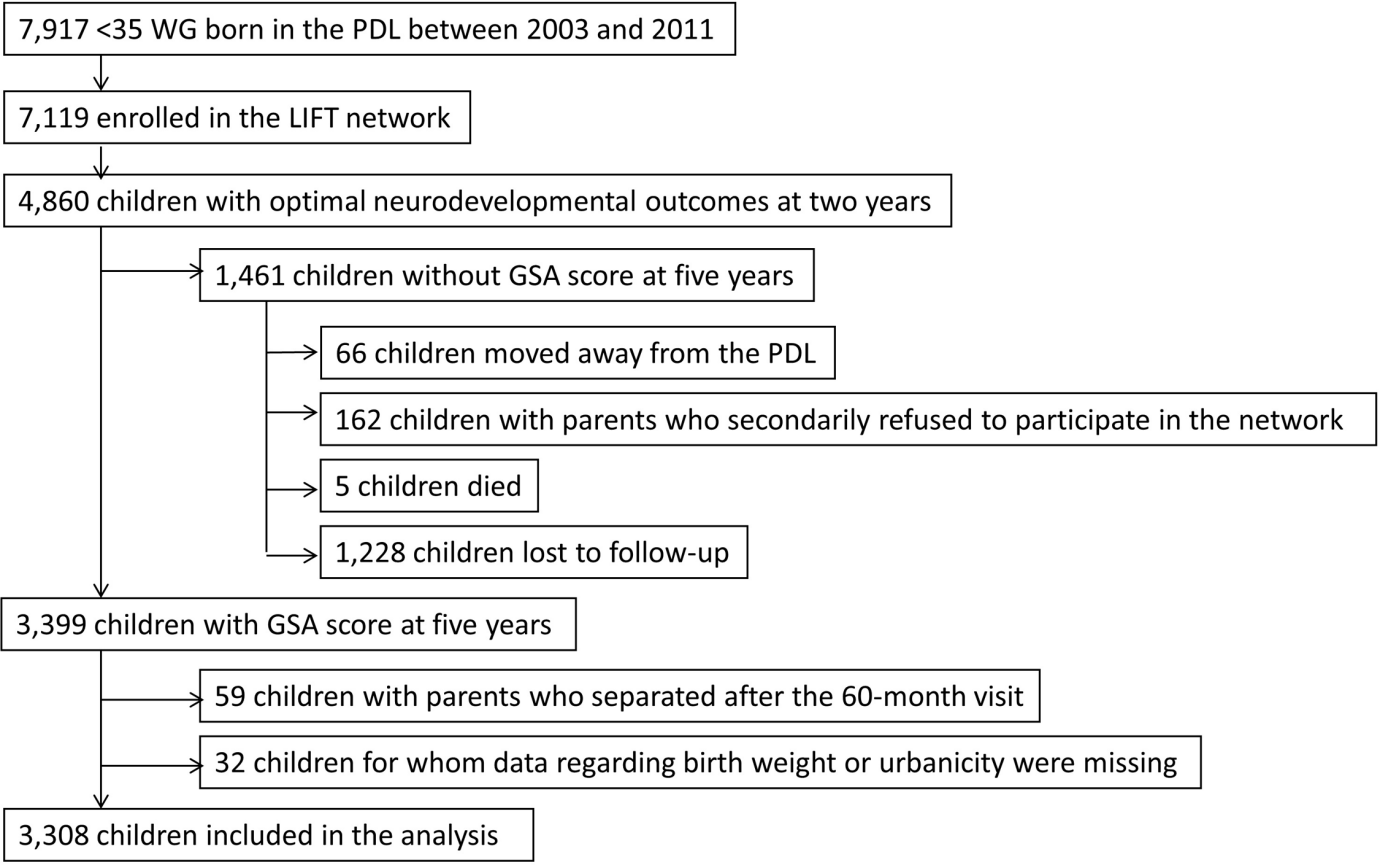
Flowchart.

Between January 2003 and December 2011, 7,119 infants born at less than 35 weeks of gestation in the Pays-de-la-Loire region were enrolled in the LIFT cohort (Fig 1). Infants who were deemed to have a non-optimal neurodevelopmental outcome at two years (n = 1,230) were excluded from the study population. Of these, 70% (n = 3,399) underwent assessment for a GSA score when they reached five years of age. The mean GSA score was 50.8 (median = 53.0) (Table 1). The proportion of parents reported to have undergone separation within five years after their child’s birth was 8.4% (n = 280), for 3.3% (n = 110) this occurred before or at two years, and for 5.1% (n = 170) this occurred between three and five years. The median timing of the visit at which the separation occurred was 36 months following the child’s birth. Of these children, 2,707 were considered further after being matched. The number of children in each group that had the same characteristics varied between 2 and 94, with a median of 15 infants.

**Table 1:** Characteristics of the overall selected and matched infants.

	Overall selected infants (n = 3308)	Matched infants (n = 2707)
Global GSA score (continuous)		
Mean (SD)	50.8 (7.6)	50.8 (7.5)
Global GSA score (categorical)		
Tercile 1: GSA [22, 49)	1,060 (32)	866 (32)
Tercile 2: GSA [49, 56)	1,109 (33.5)	917 (33.9)
Tercile 3: GSA [56, 60]	1,139 (34.4)	924 (34.1)
Parental separation		
Parents living together 5 years after the birth	3,028 (91.6)	2,439 (90.1)
Parents who underwent separation within 2 years (included) after the birth	110 (3.3)	104 (3.8)
Parents who underwent separation between 3 and 5 years following the birth	170 (5.1)	164 (6.1)
Gestational age		
32–34 weeks	2,169 (65.6)	1,953 (72.1)
28–31 weeks	931 (28.1)	692 (25.6)
24–27 weeks	208 (6.3)	62 (2.3)
Gender		
Female	1,567 (47.4)	1,254 (46.3)
Male	1,741 (52.6)	1,453 (53.7)
Having twins		
No	2,045 (61.8)	1,721 (63.6)
Yes	1,263 (38.2)	986 (36.4)
Z-score of birth weight		
>1	278 (8.4)	158 (5.8)
0–1	1,054 (31.9)	949 (35.1)
-1-0	1,224 (37)	1,040 (38.4)
<-1	752 (22.7)	560 (20.7)
Social security benefits due to low income		
No	2,990 (90.4)	2,537 (93.7)
Yes	318 (9.6)	170 (6.3)
Socioeconomic level		
Intermediate	2,355 (71.2)	2,099 (77.5)
High	953 (28.8)	608 (22.5)
Urbanicity		
Rural	1,266 (38.3)	941 (34.8)
Urban	2,042 (61.7)	1,766 (65.2)

**Fig 2:**
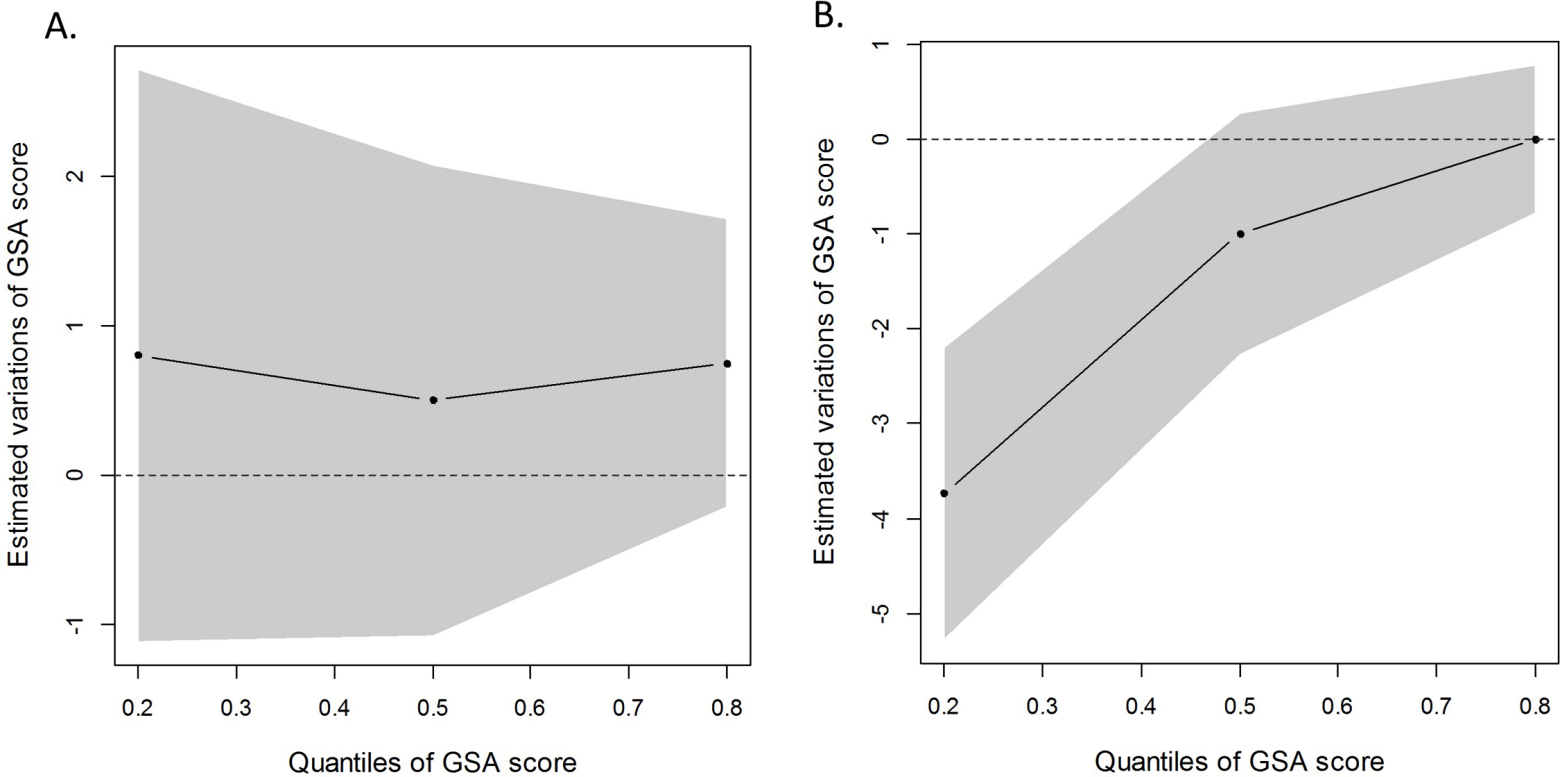
Variations in the “Global School Adaptation” (GSA) score associated with parental separation. **(**A) Separation that occurred prior to two years of age (included) (n = 104) and (B) separation that occurred between three and five years of age (n = 164), compared to children whose parents who were still living together at five years (n = 2,439). The variations were quantified based on the quantiles of the GSA (i.e. 0.2, 0.5, and 0.8).

**Fig 3:**
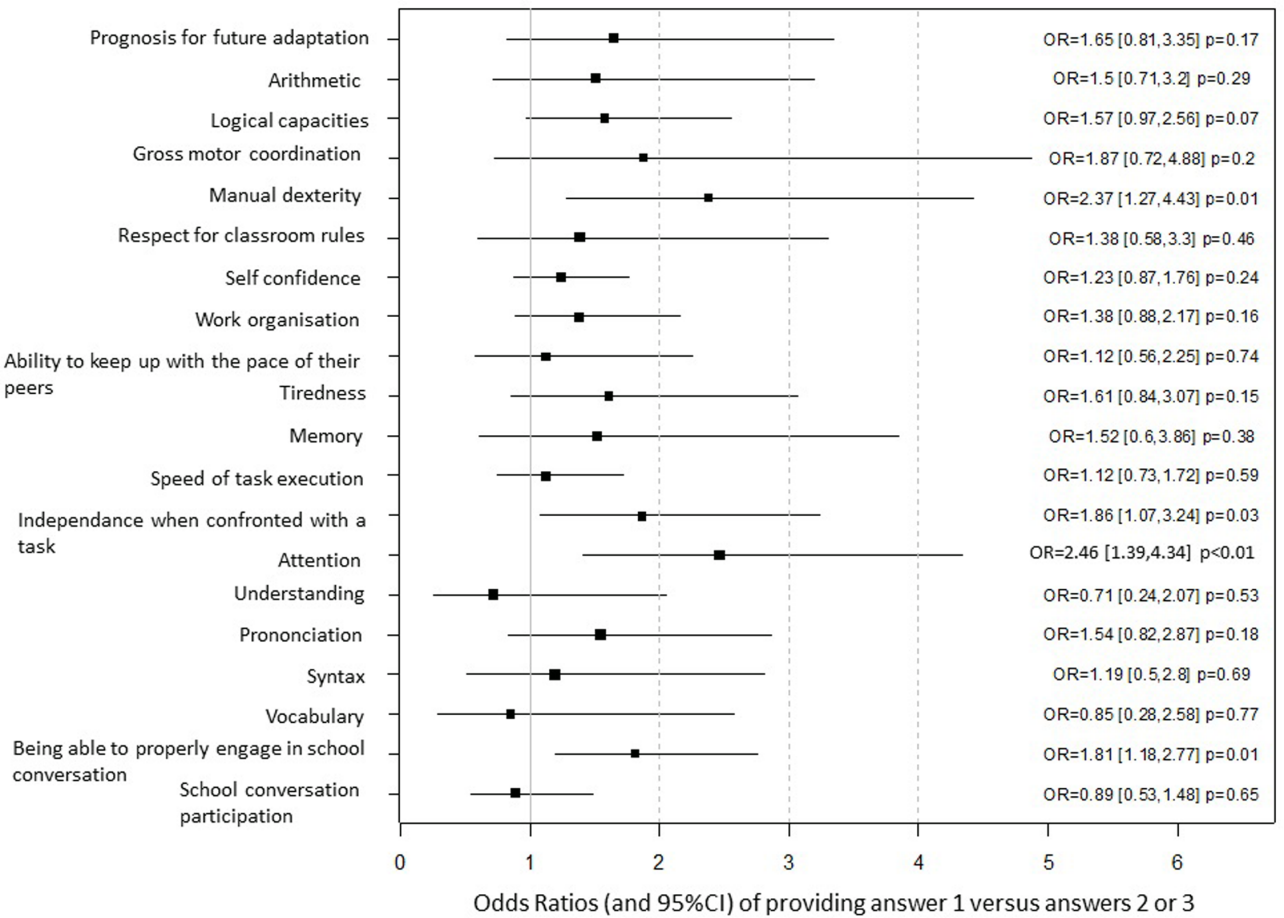
Increased risk of providing answer 1 (corresponding to the lowest score) versus Answers 2 or 3 for each item of the “Global School Adaptation” (GSA) questionnaire associated with parental separation that occurred between three and five years of age (n = 164), compared to children whose parents were still living together at five years (n = 2,439). Results were expressed as Odds Ratios (OR) with their 95% Confidence Intervals (CI).

Parental separations were associated with a lower school performance. This lower performance only occurred for children with a low GSA score (corresponding to the 20^th^ percentile) whose parents had undergone separation when the child was between 3 and 5 years of age. For these children, the decrease in performance amounted to 3.7 points (standard error = 1.54, p = 0.01) (Fig 2B). Moreover, parental separations were associated with a decrease in their ability to pay attention in the classroom (OR = 2.46 [1.39 to 4.34], p < 0.01), being able to properly engage in school conversation (OR = 1.81 [1.18 to 2.77], p = 0.01), and in terms of independence when confronted with a task (OR = 1.86 [1.07 to 3.24], p = 0.03), as well as an increase in serious difficulties with activities requiring manual dexterity (OR = 2.37 [1.27 to 4.43], p = 0.01) (Fig 3).

## Discussion

Our results indicated that, for preterm infants that had an optimal neurodevelopment at two years, parental separation was associated with a decrease in school performance at five years of age that was independent of their socioeconomic background. This decrease was only noted for parental separations that occurred between three and five years after the child’s birth when the children exhibited difficulties at school. Furthermore, parental separations were associated with a decrease in the child’s motivation, engagement, autonomy, and manual dexterity.

Parental separation only impacted children who exhibited low scholastic performances. These children faced a stressful situation at school as their poor results were exacerbated as a result of their parents being separated. Their poor scholar performances may be related to factors prior to the parental separation, such as parental conflict or lack of parental commitment to the child’s education. This would be consistent with results of previous studies that revealed a decrease in educational performance[19,20], or an increase in academic, psychological, and behavioral problems[21] prior to the parents becoming separated. Additionally, this supports the notion that divorce is a process rather than merely an event[6,22].

Moreover, only separations that occurred between three and five years were associated with decreased school performance of children at five years of age. For term infants, the same result was found regarding the age at separation. Cognitive development was found to not be affected by parental separation during the first three years of life[23]. An explanation for this could be that parental separation is unlikely to have negative consequences for children who have not yet formed a mental representation of their relationship with their parents. Moreover, these infants were likely to be too young to remember this stressful event. Furthermore, parental separation that occurred when a child is very young could have effects that were not investigated in the present study. Indeed, younger children are much more dependent on their parents for socioemotional and physical care than older children. In terms of the effect of parental separation on attachment to parents, it has been suggested that the impact appears to be more pronounced in children who are younger at the time of the family dissolution[24].

The negative consequences of parental separation comprised effects on the children’s behavior and abilities. Children whose parents had undergone a separation had more difficulty with being able to continue to pay attention, and they more readily became overwhelmed as compared to children from intact families. Consequently, their conversations at school were considered to be less appropriate by their teachers, and they had serious difficulties with activities requiring manual dexterity such as drawing or handicrafts. This result is in accordance with studies performed with term infants that have provided evidence that parental separation negatively impacts their children’s academic motivation and engagement[6].

The GSA score was used to evaluate the children’s school performances. This score, which correlates well with the IQ score, is used to detect adverse neurodevelopmental outcomes in preterm children [16]. This decrease in the GSA score corresponds with a standard deviation (SD) of 0.5. A meta-analysis carried out in regard to term infants has estimated a median decrease corresponding to 0.14 SD [5]. The more pronounced effect found in our study might be due to the vulnerability of the preterm infant population. Preterm infants could indeed be more sensitive to stressful situations such as parental separation. The difference might also be due to our statistical approach that allows an effect in a specific population to be quantified. Finally, because the median GSA score was around 40 for children with low GSA scores, we roughly estimate that parental separation is associated with a 10% diminution of the GSA score, after adjusting for children and parent/family characteristics.

As a follow-up to this study, it would be of considerable interest to investigate long-term effects of parental separation. Several studies have reported that there may be more of an impact on long-term consequences in regard to achievements and quality of life as adults than on the short-term emotional effects in children[3,25]. Another interesting aspect would be to investigate whether the effect of parental separation on school performance varies according to the parental custody and possible remarriage after separation.

A strength of this study was the large number of infants included, which allowed a high statistical power to be attained. Moreover, the exact matching allowed a very high comparability to be reached between the infants whose parents were living together and those for whom they had separated. In terms of categorical variables, the infants in these two groups had exactly the same characteristics. All of the standardized mean differences were < 0.01 after matching (S2 Fig), it can be concluded that there was an absence of bias in regard to the observed characteristics of the infants. Lastly, the use of quantile regressions allowed evidence to be obtained of a specific negative effect of parental separation for children with low GSAs. A sensitivity analysis was performed with three additional adjustment variables that can influence children’s neurodevelopment: breast fed only (yes/no), severe abnormality (affirmative, if one of the following pathologies was diagnosed: stage 3 or 4 intraventricular hemorrhaging, ventriculomegaly, periventricular leukomalacia), and the duration of oxygen supply. As the results were similar, it can be concluded that there was an absence of bias in regard to these variables (S3 Fig).

The characteristics of the children who were excluded from the study population were not comparable to those who were included (S1 Table). The excluded infants comprised those without GSA scores at five years (n = 1,461), infants whose parents separated after the 60-month visit (n = 59), or infants with incomplete medical records (n = 32). These infants were more likely to come from disrupted families with a lower socioeconomic level and who were living in rural areas, as compared to the included children. This suggests that parental separation may be a cause for non-inclusion. Children who moved to another area after their parents separated may be the most affected, as moving to another school is generally a stressful situation[26] that may be associated with a decrease in scholastic performance. The exclusion of these children might therefore lead to an underestimation of the effect of separation. Moreover, some information related to the context of the separation were not available in this study and were thus not accounted for, such as the level of parental stress, the financial sequelae, a change in residence, or the parenting roles after separation. These factors may have affected the children. Further research should thus be carried out while accounting for these factors in order to assess the causal pathway between parental separation and the cognitive performance of children.

## Conclusions

Parental separations that occurred when preterm children were between three and five years of age were associated with a decrease in the school performance when the children exhibited difficulties at school. Our results suggest that this effect could be due to a decrease in the children’s level of motivation, autonomy, and manual dexterity. In light of our findings, specific support could be given to young children who were delivered preterm who exhibit scholastic difficulties and whose parents separated when they were of elementary school-age.

## Supporting information

S1 Table Comparison of the characteristics of included and excluded children. (DOC)

S1 Fig Global School Adaptation questionnaire. Drafted by Agnes Florin, Director of the Education, Cognition, and Development laboratory and Professor of Developmental Psychology at the University of Nantes (reproduced with permission). As an example, for Question 1, the answer “just a little or not at all” received a score of 1, “moderately” received a score of 2, and “quite a lot” received a score of 3.
(DOC)

S2 Fig Standardized mean differences, before and after matching, between children whose parents were still living together at five years of age and children whose parents had undergone a separation. The right part of the figure presents the differences in percentages or means (with standard deviations) between the two populations before matching.
(TIF)

S3 Fig Variations in “Global School Adaptation” (GSA) score associated with parental separation that occurred (A) prior to two years of age (included) (n = 104) and (B) between three and five years of age (n = 164), compared to children whose parents were still living together at five years (n = 2,439). The variations were quantified according to the quantiles of the GSA (i.e. 0.2, 0.5, and 0.8). The same model as the one used for Fig 2 with three additional adjustment variables: breast fed only (yes/no), severe abnormality (affirmative, if one of the following pathologies was diagnosed: stage 3 or 4 intraventricular hemorrhage, ventriculomegaly, periventricular leukomalacia), and the duration of oxygen supply (four classes: no oxygen supply, < 28 d, between 28 and 36 d, > 36 d).
(TIF)
