# Supplementary material for: Impact of parental separation or divorce on school performance in preterm children: A population-based study
Source: PLoS One. 2018 Sep 7;13(9):e0202080. doi: 10.1371/journal.pone.0202080 (PMC6128464; doi:10.1371/journal.pone.0202080)
Supplement: S1 Table — (DOC) [file pone.0202080.s001.doc]

**S1 Table. Comparison of the characteristics of included and excluded children.**

|  | Not included* | Included | P -value |
| --- | --- | --- | --- |
| Total | 1,552 | 3,308 |  |
| Parental separation |  |  | < 0.001 |
| Parents living together 5 years after the birth | 1,264 (81.4) | 3,028 (91.5) |  |
| Parents who had undergone separation within 5 years after the birth | 288 (18.6) | 280 (8.5) |  |
| Gestational age (weeks) |  |  | 0.647 |
| 32-34 | 1,033 (66.6) | 2,169 (65.6) |  |
| 28-31 | 431 (27.8) | 931 (28.1) |  |
| 24-27 | 88 (5.7) | 208 (6.3) |  |
| Gender |  |  | 0.897 |
| Female | 739 (47.6) | 1,567 (47.4) |  |
| Male | 813 (52.4) | 1,741 (52.6) |  |
| Twins |  |  | 0.283 |
| No | 985 (63.5) | 2,045 (61.8) |  |
| Yes | 567 (36.5) | 1,263 (38.2) |  |
| Birth weight z-score |  |  | 0.259 |
| >1 | 134 (8.7) | 278 (8.4) |  |
| 0-1 | 450 (29.2) | 1,054 (31.9) |  |
| -1-0 | 582 (37.7) | 1,224 (37) |  |
| <-1 | 377 (24.4) | 752 (22.7) |  |
| Social security benefits due to low income |  |  | < 0.001 |
| No | 1309 (84.3) | 2990 (90.4) |  |
| Yes | 243 (15.7) | 318 (9.6) |  |
| Socio-economic level |  |  | < 0.001 |
| Intermediate | 1,215 (78.3) | 2,355 (71.2) |  |
| High | 337 (21.7) | 953 (28.8) |  |
| Urbanicity |  |  | < 0.001 |
| Rural | 449 (30.9) | 1,266 (38.3) |  |
| Urban | 1,004 (69.1) | 2,042 (61.7) |  |

* Excluded infants were infants without a GSA score at 5 years (n=1,461), infants whose parents underwent a separation after the 60-month visit (n=59), or infants with incomplete medical records (n=32).
