# Supplementary material for: Impact of parental separation or divorce on school performance in preterm children: A population-based study
Source: PLoS One. 2018 Sep 7;13(9):e0202080. doi: 10.1371/journal.pone.0202080 (PMC6128464; doi:10.1371/journal.pone.0202080)
Supplement: S1 Fig — Drafted by Agnes Florin, Director of the Education, Cognition, and Development laboratory and Professor of Developmental Psychology at the University of Nantes (reproduced with permission). As an example, for Question 1, the answer “just a little or not at all” received a score of 1, “moderately” received a score of 2, and “quite a lot” received a score of 3. (DOC) [file pone.0202080.s002.doc]

**S1 Fig:** **Global School Adaptation questionnaire.** Drafted by Agnes Florin, director of the Education, Cognition and Development laboratory and professor of developmental psychology at the University of Nantes (reproduced with permission). As an example, for the question 1, the answer “just a little or not at all” received a score of 1, “moderately” received a score of 2, and “quite a lot” received a score of 3.
