# Supplementary figures and images for: Impact of parental separation or divorce on school performance in preterm children: A population-based study
Source: PLoS One. 2018 Sep 7;13(9):e0202080. doi: 10.1371/journal.pone.0202080 (PMC6128464; doi:10.1371/journal.pone.0202080)

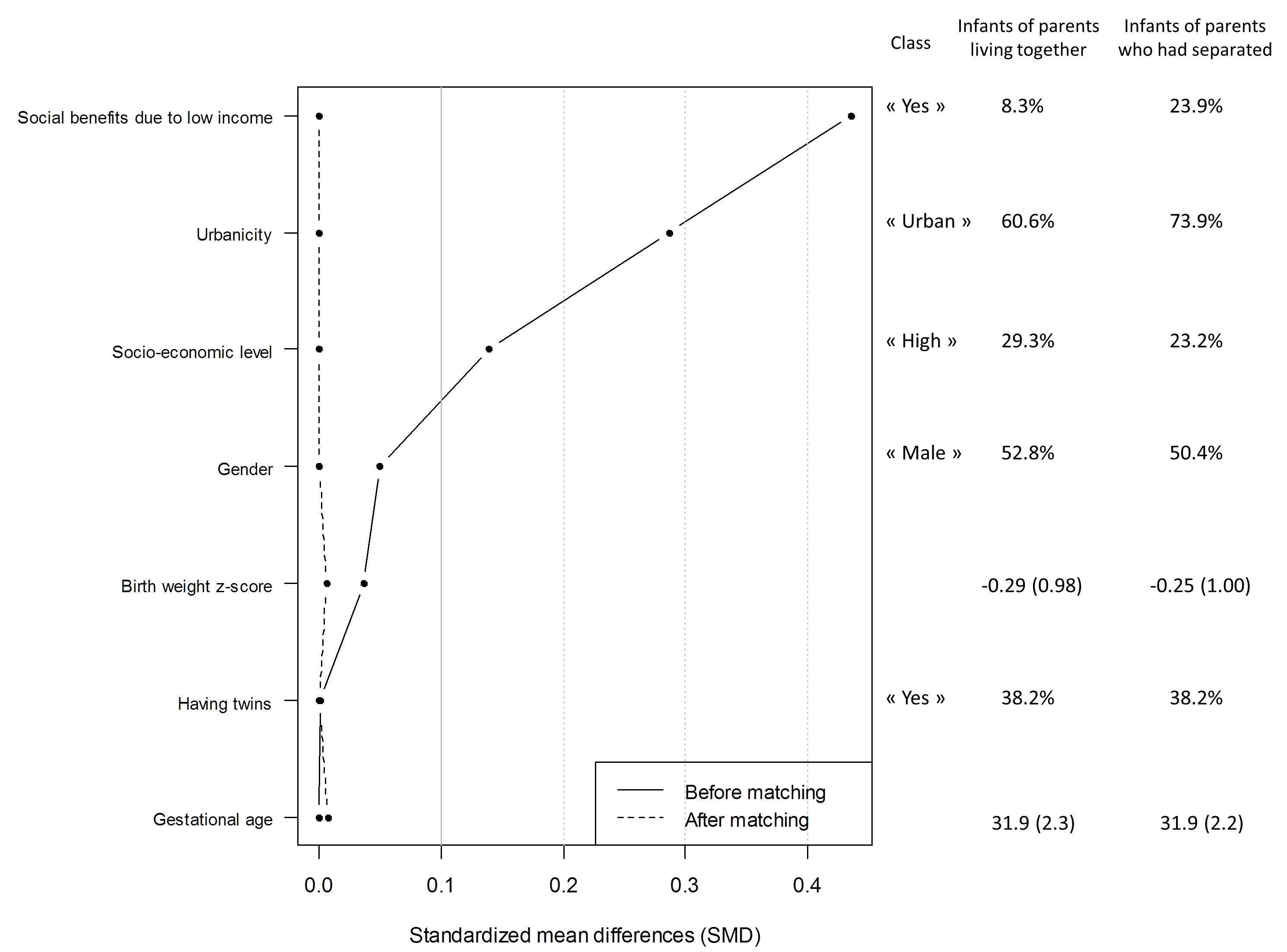

Supplement: S2 Fig — The right part of the figure presents the differences in percentages or means (with standard deviations) between the two populations before matching. (TIF) [file pone.0202080.s003.tif]

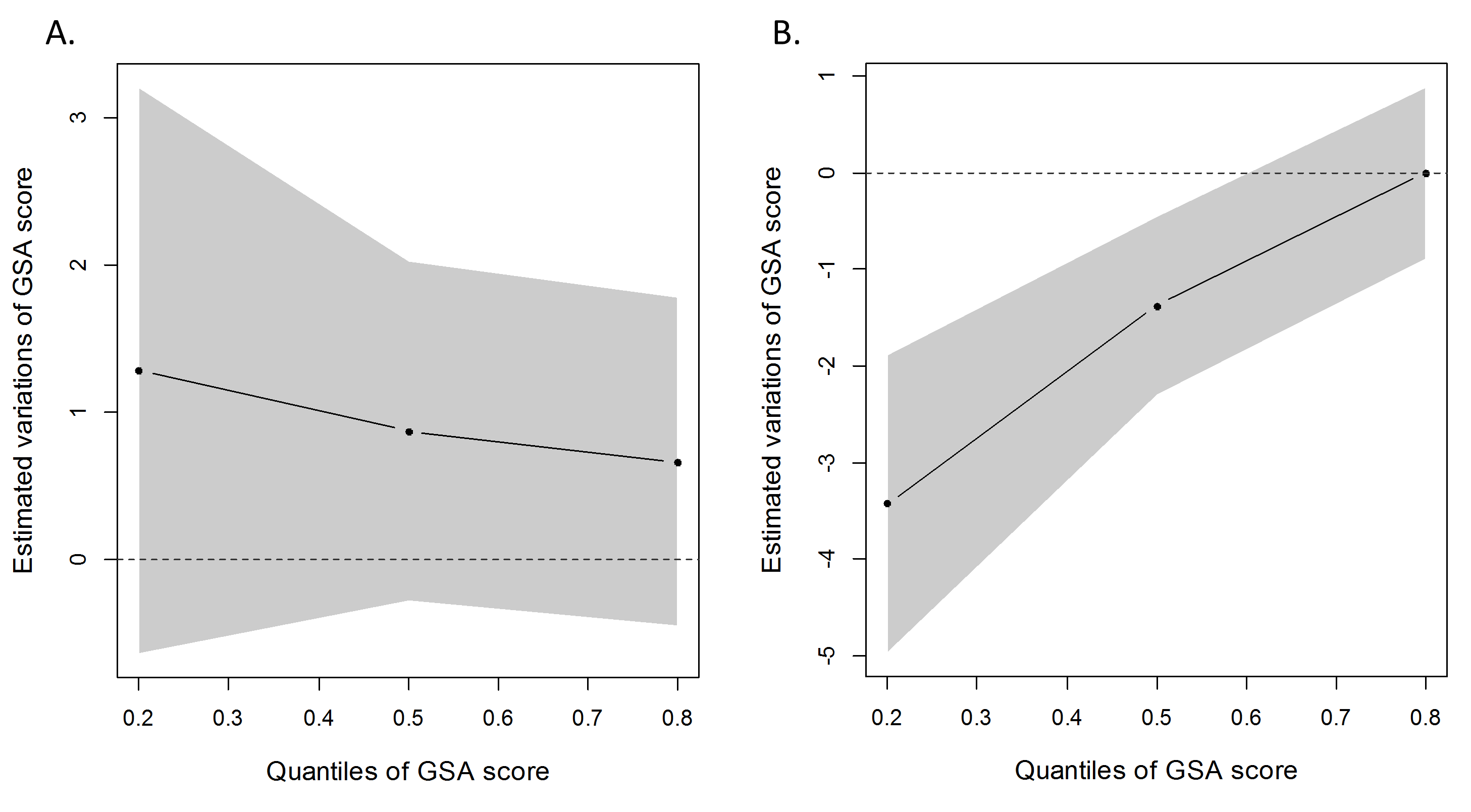

Supplement: S3 Fig — The variations were quantified according to the quantiles of the GSA (i.e. 0.2, 0.5, and 0.8). The same model as the one used for Fig 2 with three additional adjustment variables: breast fed only (yes/no), severe abnormality (affirmative, if one of the following pathologies was diagnosed: stage 3 or 4 intraventricular hemorrhage, ventriculomegaly, periventricular leukomalacia), and the duration of oxygen supply (four classes: no oxygen supply, < 28 d, between 28 and 36 d, > 36 d). (TIF) [file pone.0202080.s004.tif]
